# The structure of selective dinucleotide interactions and periodicities in D melanogaster mtDNA

**DOI:** 10.1186/0717-6287-47-18

**Published:** 2014-05-23

**Authors:** Carlos Y Valenzuela

**Affiliations:** Programa de Genética Humana, ICBM, Facultad de Medicina, Universidad de Chile, Independencia 1027, Santiago, Chile

**Keywords:** Dinucleotides, DNA periodicity, Periodicity structure, Selective interactions, Theories of evolution

## Abstract

**Background:**

We found a strong selective 3-sites periodicity of deviations from randomness of the dinucleotide (DN) distribution, where both bases of DN were separated by 1, 2, K sites in prokaryotes and mtDNA. Three main aspects are studied. I) the specific 3 K-sites periodic structure of the 16 DN. II) to discard the possibility that the periodicity was produced by the highly nonrandom interactive association of contiguous bases, by studying the interaction of non-contiguous bases, the first one chosen each I sites and the second chosen J sites downstream. III) the difference between this selective periodicity of association (distance to randomness) of the four bases with the described fixed periodicities of base sequences.

**Results:**

I) The 16 pairs presented a consistent periodicity in the strength of association of both bases of the pairs; the most deviated pairs are those where G and C are involved and the least deviated ones are those where A and T are involved. II) we found significant non-random interactions when the first nucleotide is chosen every I sites and the second J sites downstream until I = J = 76. III) we showed conclusive differences between these internucleotide association periodicities and sequence periodicities.

**Conclusions:**

This relational selective periodicity is different from sequence periodicities and indicates that any base strongly interacts with the bases of the residual genome; this interaction and periodicity is highly structured and systematic for every pair of bases. This interaction should be destroyed in few generations by recurrent mutation; it is only compatible with the Synthetic Theory of Evolution and agrees with the Wright’s adaptive landscape conception and evolution by shifting balanced adaptive peaks.

**Electronic supplementary material:**

The online version of this article (doi:10.1186/0717-6287-47-18) contains supplementary material, which is available to authorized users.

## Background

We undertook a research to test evolutionary theories by studying the relationships among nucleotides independently of their allocation in any nucleotide sequence (periodic or non-periodic), coding or non-coding for protein synthesis or its regulation, centromeres or telomeres and repeated DNA sequences dispersed or in tandem. Our position was that any structured or organized DNA sequence or relationship among nucleotides, maintained during thousands or millions of cell generations, was necessarily a proof of the Synthetic Theory of Evolution (STE), given that this maintenance is impossible by random or neutral evolutionary processes with recurrent forward and backward mutation; the addition of purifying or weak selection does not change this condition [1, 2]. Thus our method of study interactions between or among nucleotides should prevent, avoid or be independent of any structured DNA sequence so as to inform if any non sequential organization was present. Our aim was to determine the strength of non-random (neutral versus selective) association of two nucleotides or bases taken at random from a genome, chromosome or DNA segments according to the number of nucleotide sites between both independently of any functional or structured sequence at which they belong. Physical interactions or correlations have been found more recently [3]. We studied all the possible dinucleotides of a genome or genome segment classified only by the separation in number (K) of nucleotide sites between both bases, K in this case goes from 0 (contiguous bases) to N-K-1, where N is the number of sites of a chromosome. Then we obtain a total of [(N-K)(N-K-1)/2] dinucleotides (pairs); but each series for each K yields (N-K-1) pairs; in each series the first base runs from the initial base to the (N-K-1)_th_ base and the second base runs from the (initial + K +1)_th_ base to the N_th_ base. Then our exigency of independence of DNA structures is satisfied because the first nucleotide (that one located upstream) runs (equally) over all the sites of the chromosome excepting that one of the second nucleotide, and the second nucleotide (that one located downstream) also runs over all the chromosome sites excepting that one of the first nucleotide, for each independent series of pairs (remark that nucleotides are re-sampled for each K-series). Thus both nucleotides are equally sampled from all the DNA structures and yield an average of these structured or non-structured sequences. Thus we obtain series of pairs classified according to K [0 (contiguous), 1, 2, 3…K] sites. For each series of the total dinucleotides with K separation we study the expected (Exp) and observed (Obs) number of the 16 possible pairs and obtain the stochastic continuous random variable known as the χ^2^ value [sum of (Obs-Exp)^2^/Exp] and the selection coefficient of each pair estimated by [(Obs_i_-Exp_i_)/Exp_i_; i from 1 to 16] expressed with positive and negative values. The expected number of pairs is obtained after estimating the base frequencies fA, fT, fG and fC found in the chromosome or DNA segment. We assumed that these frequencies were the random (neutral) expected base frequencies of this DNA maintained for millions generations. Then we estimate the expected pair frequency by the corresponding product of frequencies, as for example fAA = fAxfA; fCG = fCxfG, etc. The expected number of a pair is its frequency times the total number of pairs separated by K sites. The procedure is similar as taking a big random sample of dinucleotides, anywhere in the chromosome, classifying them by the number of sites that separate both bases and ordering by assigning the first nucleotide upstream; also it is equivalent to choose two random samples of the same size one for the first nucleotide and the other for the second nucleotide and then matching the bases in dinucleotides, excluding the equal nucleotides, and classifying them by the number of sites between the first (upstream) and the second base. The facility to obtain genomes allows us to work with the total number of dinucleotides with separations until K = 3,000; it is not necessary to reach K = N-2. We must remark that since the first base is at any site of the chromosome, independently of its allocation in periodic or non periodic regions, coding or non coding regions, and for each first base the second base goes also through any site of the chromosome (in the whole set of K series), this procedure destroys any relationship of nucleotide sequence, periodic or non-periodic, coding or non coding regions for protein synthesis or regulation. The site at which the collection of pairs begins, the sense, up or downstream, at which it moves are irrelevant for the result, provided that we assign the first base that one is upstream. We shall see that even this criterion of conserving the 3′–5′ sense is not strictly necessary because the base to base interaction follows the expected correlation due to base complementariness in a great proportion, as we shall show. Thus the collections of pairs of each K series may begin at different sites and proceed up or downstream or both alternately and the result is the same, even though the samples of pairs do not include their total.

Our first expectancy with this method, similar to a machine of chromosome grinding and nucleotides stirring, was that nothing different from randomness, because the machine produces an average of any possible association, had to be obtained. Our surprise was enormous because the method uncovered strong pan-selective interactions between the bases of dinucleotides in complete genomes or DNA segments [4–7]. The conclusion was that any base is strongly co-adapted with any other base (residual genome) of the chromosome. It is important to remark that our aim was never searching for sequence periodicities; on the contrary the method destroys them. We found among these selective interactions, as serendipity discovered by undergraduate students, a significant periodicity of the χ^2^ value (not of DNA sequences) for non-random dinucleotide bases’ association in prokaryotes and mtDNA [1, 4–9]. This stochastic (the χ^2^ value is a random variable whose value varies stochastically) periodicity is completely different from sequence DNA periodicities that are well documented and have been assumed to happen in relation to DNA coiling and hypercoiling, sites of action of enzymes, DNA binding to proteins, histones or histone-like proteins and other DNA properties [10–27]. Among these sequence periodicities, three nucleotides periodicity in prokaryotes, and several periodicities, included those with peaks at 84 or 167 bp in human chromosome 21 and 22, in eukaryotes have been described [17, 21, 22]. However, these are periodicities of DNA sequences of bases or sets of bases repeated in tandem or dispersed along with the genome. They have been studied by Fourier series, autocorrelations or latent periodicities [12, 13, 15, 17, 22, 28]. All these studies search for finding a non random periodic repetition of a sequence with a known period or for finding periodic sequences defined by textual strings of length J. This is the aim in the studies of latent periodicities that define strings and search for the specific probability of a base to fit its position in this period of length J [28]. In our study the separation of K sites between both bases of a dinucleotide has nothing related to the j position within the period. For our method the sequence between both sites not only is irrelevant but it is not considered for the analyses, and since dinucleotides belong to any segment, it is averaged in the total set of different segments and in the different K series. It is very important to remark a different epistemological perspective of our studies that assume a non-theoretical background except neutral or random distribution of nucleotides included in the standard theories of evolution. No previous knowledge of DNA structure is assumed; they take the DNA sequence and study the nucleotide relationships in sequences of nucleotide as a series of four different colored balls in a file of balls; after obtaining results it is difficult or impossible to compare with theoretical based studies. We found that this distribution was the Bose-Einstein distribution [1, 4, 29].

So defined, our periodicities, found as a serendipity, compare completely random with actual dinucleotide totals to test critically the STE with the Neutral (or Nearly-Neutral) Theory of Evolution (NTE or N-NTE, respectively). The periods of these new stochastic periodicities are produced by variation of the value of the significance of deviation from randomness, which are a marginal part of the huge significant interactions among all the bases of a genome [1, 4–8]. All the previous periodicities are sequence periodicities, where the period corresponds to a linear nucleotide sequence (string), and they are located in functional or specific parts of genomes. Our periodicities are not periodicities of sequence of bases nor are their period a sequence of bases. These new periodicities are periodicities of the magnitude and structure of deviations from the random expected dinucleotides’ distribution or the strength of non-random association of the bases of dinucleotides, and are a property of the whole genome and allocated anywhere in it.

In sequence periodicities the χ^2^ test is used as a tool to demonstrate that a latent periodicity does exist; in our periodicities the χ^2^ test is not used to demonstrate anything, it is by itself or it includes a periodicity that is by no means a latent, cryptic, invisible, hidden or non-evident relationship; it is seen at the first sight with probabilities often lower than 10^−30^ in this mtDNA with no more than 20,000 bp where latent periodicities, if they are present, are very difficult to be evidenced (no references were found on this subject). The physical image of the total spectrum of bases of this mtDNA has been previously analyzed and presented [6], and no periodicity was found excepting segments of AT-AT-AT… Moreover, this mtDNA codes in both strands for tDNA, rDNA, mDNA and for replication DNA segments; thus its less than 20,000 bp includes mostly meaningful segments and when a set of pairs with separations longer than 500 sites between both bases are collected, most of these pairs shall have one base in one meaningful segment and the other base in another different meaningful segment. These χ^2^ value periodicities are by constitution periodicities of the stochastic strength of non-random association (attraction or repulsion) of both bases separated by K sites. Moreover, the structure of these periodicities may be constructed with different bases in different organisms and within an organism in different parts of its genome [4–7]. This is a periodicity for the magnitude and sign (+ or -) of selection and the distance to non-randomness of the distribution of the 16 possible dinucleotides. Thus it is by constitution, a selective periodicity of the strength of association of bases among the 16 dinucleotides (remember our initial and present aim was and is to test critically the STE versus the NTE-N-NTE [1, 4–8, 29–34]).

The three aims of this article are: I) to describe the specific structure of the selective interaction and stochastic periodicity of each of the 16 dinucleotides in *Drosophila melanogaster* mtDNA; II) to demonstrate that the periodicity is not due to the high non-random interaction of contiguous or closely related nucleotides; III) to establish differences between this selective periodicity and the sequence periodicities previously described in literature. Here the genome, genome segments, dinucleotides or set of dinucleotides are taken as phenotypes. Our previous studies showed that these periodicities were intricate entangled wools; the present study of the structure of deviations of the 16 dinucleotides intends to find the tip of the thread to begin to disentangle these wools.

## Results and Discussion

### Searching for the structure and origin of the stochastic periodicity

Tables 1 and 2 present the periodicity structure of the contribution of each pair to the χ^2^_9_ value, for the 16 pairs. Table 1 refers to the case when the first base is Adenine and Thymine and Table 2 when they are Guanine and Cytosine. In both tables we show the previous result of significant total χ^2^_9_ until 23 K (remember that K = 1 indicates contiguous bases, or 0 separation sites). Our previous studies showed that χ^2^_9_ values are significant until 2,000 K and more [6–9]; with these separations all the pairs have the first base in one meaningful DNA segment and the second base in another meaningful segment (the largest meaningful segment is a mDNA near 1,600 bp). Sequence periodicities are discarded because they cannot yield these huge significant values in this small mtDNA. The structure of periodicities is shown by the χ^2^_1_ values of each pair. In Table 1 we see that the 8 pairs (4 for A and 4 for T) continue to be periodically significant until K = 24 with the exception of A-T and T-A whose significant values decrease substantially over K = 11. In Table 2 we see that the 8 pairs (4 for Guanine and 4 for Cytosine) have significant periodicities until K = 24. It is evident that G and C deviate more significantly than A and T and their periodicities are significant for larger Ks. Tables 1 and 2 show also that the sign of deviations from randomness, estimated by the selective coefficient [(Obs-Exp)/Exp], is not randomly distributed. In 24 Ks we should find 12 + and 12 – signs if they are randomly distributed. For A-A pairs we found 20+ and 4- signs (binomial P = 0.00014); A-T pairs showed 12+ and 12-, the most probable result (P = 0.58); in A-G pairs there were 5+ and 19- pairs (P = 0.00077); in A-C pairs 5+ and 19– (P = 0.00077). In T-A pairs 15+ and 9- (P = 0.1537); T-T pairs 21+ and 3- (P = 0.000018); T-G pairs 9+ and 15- (P = 0.1537); T-C pairs 4+ and 20- (P = 0.00014). For G-A pairs 4+ and 20- (P = 0.00014); G-T pairs 7+ and 17- pairs (P = 0.011); G-G pairs 21+ and 3- (P = 0.000018); G-C pairs 18+ and 6- (P = 0.0033). In C-A pairs 4+ and 20- (P = 0.00014); C-T pairs 5+ and 19- (P = 0.00077); C-G pairs 16+ and 8- (P = 0.032); C-C pairs 22+ and 2- (P = 0.0000015). The C-G pair show a clear periodicity - + +, − + +, − ++… where the positive signs belong to highly significant χ^2^ values, while the negative sign is seen in lower or non significant χ^2^ values. Thus, we uncover the tip of the thread for this periodicity: C tends to associate with G very stronger than at random (attraction?) when (K-1) sites separate both bases; C associates to G less frequently than at random (repulsion?) when K sites separate both bases; C tends again to associate to G stronger than at random (but less significantly than in the case of K-1 sites between them) when (K + 1) sites separate both bases.

**Table 1:** **Selective deviation of pairs measured by the χ** ^**2**^_**1**_ **contribution to the χ** ^**2**^_**9**_ **test and their sign of deviation (first base A and T)**

1° B		Adenine				Thymine			
2° B		A	T	G	C	A	T	G	C
SEP	Tχ^2^_9_	Contribution of pairs (χ^2^_1_) to the total χ^2^_9_ value							
1	485.1	10.5+	0.2-	0.6-	25.4	4.7-	27.8+	10.5-	10.9-
2	94.3	0.3+	0.5+	6.0-	0.2-	0.0+	2.1+	1.6-	3.6-
3	405.0	4.0+	0.8+	5.4-	14.4-	3.4+	4.6+	23.0-	14.7-
4	114.2	22.0+	14.0-	0.3+	6.1-	10.8-	14.7+	0.0+	1.2-
5	46.1	0.5-	5.9+	7.6-	1.1-	0.2+	0.7-	0.0+	0.5+
6	380.5	10.2+	3.3+	31.9-	26.8-	0.2+	0.8+	4.5-	0.6-
7	86.8	5.1-	2.6+	1.0+	0.2+	13.5+	3.6-	0.1-	12.1-
8	36.7	0.8+	0.0-	1.1-	0.5-	0.8-	1.9+	2.9-	0.3+
9	375.3	3.8+	1.9+	11.2-	14.6-	4.7+	0.7+	23.9-	3.3-
10	75.7	0.4+	0.3-	0.2-	0.1+	1.1+	0.6+	0.0-	12.0-
11	49.3	0.9-	4.3+	6.4-	0.0-	1.2+	1.2-	0.2-	0.1+
12	367.1	11.6+	0.0+	22.5-	7.8-	0.0+	7.3+	8.8-	9.2-
13	65.3	0.0-	0.0+	0.0-	0.0+	0.7+	0.1+	0.4+	7.9-
14	69.9	5.1+	0.2-	13.0-	0.2-	1.7-	0.1+	0.5+	1.8+
15	310.0	13.4+	0.1-	17.4-	10.1-	0.2+	4.4+	9.5-	6.1-
16	60.1	1.1+	0.8-	0.7+	1.1-	0.4+	0.0+	0.0+	3.2-
17	51.7	3.1+	0.0-	12.4-	0.1-	0.7-	0.7+	0.2+	0.1-
18	322.2	22.4+	2.4-	16.4-	9.0-	1.6-	14.8+	11.6-	4.8-
19	65.8	3.3+	4.7-	1.5+	0.2-	0.1-	2.0+	0.0+	5.1-
20	40.6	0.2+	0.6+	12.7-	0.5+	0.0+	0.0+	0.5+	0.5-
21	388.0	20.1+	1.5-	17.8-	8.9-	0.4-	15.1+	7.9-	16.4-
22	53.2	1.8+	2.0-	0.1+	0.0-	0.0+	1.2+	0.0-	5.1-
23	56.3	0.7+	0.2+	17.4-	1.0+	0.5-	0.1+	2.8+	0.3-
24	303.5	12.2+	0.0+	22.4-	11.1-	0.0+	4.4+	7.6-	3.5-

SEP = N° of sites of separation. T χ^2^_9_ = Total χ^2^_9_ published previously. + or - = more or less observed than expected pairs, respectively.

**Table 2:** **Selective deviation of pairs measured by the χ** ^**2**^_**1**_ **contribution to the χ** ^**2**^_**9**_ **test and their sign of deviation (first base G and C)**

1° B		Guanine				Cytosine			
2° B		A	T	G	C	A	T	G	C
SEP	Tχ^2^_9_	Contribution of pairs (χ^2^_1_) to the total χ^2^_9_ value							
1	485.1	1.3+	91.2-	124.0+	50.1+	10.5-	1.9-	2.6-	113.0+
2	94.3	0.2+	1.0-	2.9+	0.2+	2.7-	11.9-	36.3+	24.8+
3	405.0	17.7-	10.8-	115.7+	33.4+	16.5-	10.2-	24.7+	105.7+
4	114.2	4.7-	0.5-	1.3+	23.1+	1.2-	0.3+	5.1-	9.2+
5	46.1	1.0+	2.0-	1.5+	0.1-	0.2-	4.2-	20.1+	1.0+
6	380.5	14.8-	16.2-	139.4+	31.5+	15.9-	3.6-	29.8+	51.0+
7	86.8	5.8-	0.5-	0.0-	38.2+	0.5-	1.1+	2.3-	0.3+
8	36.7	0.6-	0.0+	2.6+	0.0-	0.4+	7.3-	17.3+	0.3+
9	375.3	20.9-	11.1-	148.7+	28.8+	18.6-	2.5-	35.5+	45.1+
10	75.7	15.5-	0.0-	8.0+	34.4+	0.0+	0.0-	1.6-	1.5+
11	49.3	0.3-	0.2+	0.6+	0.2-	0.0+	5.7-	28.1+	0.0-
12	367.1	21.0-	15.2-	144.0+	44.6+	9.8-	4.0-	26.4+	34.8+
13	65.3	13.1-	0.1+	1.1+	34.4+	2.7+	1.2-	3.4-	0.2+
14	69.9	0.3-	2.6+	0.1-	3.4-	2.2-	1.2-	37.5+	0.0-
15	310.0	21.6-	5.8-	77.5+	43.6+	18.6-	2.1-	48.1+	31.5+
16	60.1	10.2-	0.0+	0.2+	33.6+	0.3-	1.8+	6.2-	0.4+
17	51.7	0.4-	0.0-	1.8+	0.0+	1.9-	2.2-	26.5+	1.7+
18	322.2	16.0-	6.1-	91.4+	22.4+	13.1-	5.8-	44.8+	39.8+
19	65.8	11.2-	0.7+	0.0-	28.1+	0.0-	0.7+	7.5-	0.6+
20	40.6	0.0-	0.0-	2.3+	0.6-	0.6-	2.2-	19.5+	0.4+
21	388.0	23.4-	12.7-	93.4+	73.1+	13.6-	4.7-	33.7+	45.2+
22	53.2	8.0-	0.2-	1.8+	29.0+	0.2-	1.2+	2.5-	0.0+
23	56.3	0.1+	0.4+	0.0+	4.0-	0.3-	3.9-	24.2+	0.5+
24	303.5	16.0-	6.1-	91.7+	12.4+	13.6-	5.8-	46.1+	40.6+

Nomenclature as in Table 1.

If we see attentively the χ^2^_1_ values (increases and decreases) we observe a tendency towards periods of three sites in all the pairs. This can be study easily by the first and second discrete derivative (FD and SD, respectively) of the χ^2^ values (delta χ^2^) they are obtained by the subtraction of the n_th_ value to the (n + 1)_th_ value, and this resulting value (the numerator) divided by the difference in the corresponding Ks (delta K, the denominator) that is 1 and can be omitted. Caution! The signs of these differences are completely different from the signs of Tables 1 and 2. Table 3 and 4 show the value and sign of the first and the sign of the second derivatives for Tables 1 and 2, respectively. The first row (K = 1) is omitted; it does not have derivative.

**Table 3:** **First (F) and second (S) discrete derivatives (D) of the χ** ^**2**^_**1**_ **value, with their – or + sign, for non random periodic deviations of pairs shown in Table** 1 **(First nucleotide A and T)**

1° B	Adenine				Thymine			
2° B	A	T	G	C	A	T	G	C
SEP	FD with its sign and sign of the SD							
2	10.2-	0.3+	5.4+	25.2-	4.7-	25.7-	8.9-	7.3-
3	3.7+ +	0.3+ +	0.6- -	14.2+ +	3.4+ +	2.5+ +	21.4+ +	11.1+ +
4	18.0+ +	13.2+ +	5.1- -	8.3- -	7.4+ +	10.1+ +	23.0- -	13.5- -
5	21.5- -	8.1- -	7.3+ +	5.0- +	10.6- -	14.0- -	0.0 ± +	0.7- +
6	9.7+ +	2.6- +	24.3+ +	25.7+ +	0.0 ± +	0.1+ +	4.5+ +	0.1+ +
7	5.1- -	0.7- +	30.9- -	26.6- -	13.3+ +	2.8+ +	4.4- -	11.5+ +
8	4.3- +	2.6- -	0.1+ +	0.3+ +	12.7- -	1.7- -	2.8+ +	11.8- -
9	3.0+ +	1.9+ +	10.1+ +	14.1+ +	3.9+ +	1.2- +	21.0+ +	3.0+ +
10	3.4- -	1.6- -	11.0- -	14.5- -	3.6- -	0.1- +	23.9- -	8.7+ +
11	0.5+ +	4.0+ +	6.2+ +	0.1- +	0.1+ +	0.6+ +	0.2+ +	11.9- -
12	10.7+ +	4.3- -	16.1+ +	7.8+ +	1.2- -	6.1+ +	8.6+ +	9.1+ +
13	11.6- -	0.0 ± +	22.5- -	7.8- -	0.7+ +	7.2- -	8.4- -	1.3- -
14	5.1+ +	0.2+ +	13.0+ +	0.2+ +	1.0+ +	0.0 ± +	0.1+ +	6.1- -
15	8.3+ +	0.1- -	4.4+ −	9.9+ +	1.5- -	4.3+ +	9.0+ +	4.3+ +
16	12.3- -	0.7+ +	16.7- -	9.0- -	0.2+ +	4.4- -	9.5- -	2.9- -
17	2.0+ +	0.8- -	11.7+ +	1.0- +	0.3+ +	0.7+ +	0.2+ +	3.1- -
18	19.3+ +	2.4+ +	4.0+ −	8.9+ +	0.9+ +	14.1+ +	11.4+ +	4.7+ +
19	19.1- -	2.3+ −	14.9- -	8.8- -	1.5- -	12.8- -	11.6- -	0.3+ −
20	3.1- +	4.1- -	11.2+ +	0.3+ +	0.1- +	2.0- +	0.5+ +	4.6- -
21	19.9+ +	0.9+ +	5.1+ −	8.4+ +	0.4+ +	15.1+ +	7.4+ +	15.9+ +
22	18.3- -	0.5+ −	17.7- -	8.9- -	0.4- -	13.9- -	7.9- -	11.3- -
23	1.1- +	1.8- -	17.3+ +	1.0+ +	0.5+ +	1.1- +	2.8+ +	4.8- +
24	11.5+ +	0.2- +	5.0+ −	10.1+ +	0.5- -	4.3+ +	4.8+ +	3.2+ +

**Table 4:** **First (F) and second (S) discrete derivatives (D) of the χ2 value, with their – or + sign, for non random periodic deviations of pairs shown in Table** 2 **(First nucleotide G and C)**

1° B	Guanine				Cytosine			
2° B	A	T	G	C	A	T	G	C
SEP	FD with its sign and sign of the SD							
2	1.1-	90.2-	121.1-	49.9-	7.8-	10.0+	33.7+	88.2-
3	17.5+ +	9.8+ +	112.8+ +	33.2+ +	13.8+ +	1.7- -	11.6- -	80.9+ +
4	13.0- -	10.3- -	114.4+ −	10.3- -	15.3- -	9.9+ −	19.6- -	96.5- -
5	3.7- +	1.5+ +	0.2+ +	23.0- -	1.0- +	3.9+ +	15.0+ +	8.2- +
6	13.8+ +	14.2+ +	137.9+ +	31.4+ +	15.7+ +	0.6- -	9.7+ −	50.0+ +
7	9.0- -	15.7- -	139.4- -	6.7+ −	15.4- -	2.5- -	27.5- -	50.7- -
8	5.2- +	0.5- +	2.6+ +	38.2- -	0.1- +	6.2+ +	15.0+ +	0.0 ± +
9	20.3+ +	11.1+ +	146.1+ +	28.8+ +	18.2+ +	4.8- -	18.2+ +	44.8+ +
10	5.4- -	11.1- -	140.7- -	5.6+ −	18.6- -	2.5- +	33.9- -	43.6- -
11	15.2- -	0.2+ +	7.4- +	34.2- -	0.0 ± +	5.7+ +	26.5+ +	1.5- +
12	20.7+ +	15.0+ +	143.4+ +	44.4+ +	9.8+ +	1.7- -	1.7- -	34.8+ +
13	7.9- -	15.1- -	142.9- -	10.2- -	7.1- -	2.8- -	23.0- -	34.6- -
14	12.8- -	2.5+ +	1.0+ +	31.0- -	0.5- +	0.0 ± +	34.1+ +	0.2- +
15	21.3+ +	3.2+ +	77.4+ +	40.2+ +	16.4+ +	0.9+ +	10.6+ −	31.5+ +
16	11.4- -	5.8- -	77.3- -	10.0- -	18.3- -	0.3- -	41.9- -	31.1- -
17	9.8- +	0.0 ± +	1.6+ +	33.6- -	1.6+ +	0.4+ +	20.3+ +	1.3+ +
18	15.6+ +	6.1+ +	89.6+ +	22.4+ +	11.2+ +	3.6+ +	18.3+ −	38.1+ +
19	4.8- -	5.4- -	91.4- -	5.7+ −	13.1- -	5.1- -	37.3- -	39.2- -
20	11.2- -	0.7- +	2.3+ +	27.5- -	0.6+ +	1.5+ +	12.0+ +	0.2- +
21	23.4+ +	12.7+ +	91.1+ +	72.5+ +	13.0+ +	2.5+ +	14.2+ +	44.8+ +
22	15.4- -	12.5- -	91.6- -	44.1- -	13.4- -	3.5- -	31.2- -	45.2- -
23	7.9- +	0.2+ +	1.8- +	25.0- +	0.1+ +	2.7+ +	21.7+ +	0.5+ +
24	15.9+ +	5.7+ +	91.7+ +	8.4+ +	13.3+ +	1.9+ −	21.9+ +	40.1+ +

As expected a fascinating result appears. Two previous considerations are necessary for this analysis: I) the largest χ^2^ values are given in the first, second and third Ks, thus these values may be largely influenced by contiguousness (see next analyses), thus sign periodicity should be searched in larger Ks; II) this is a stochastic periodicity so the larger the χ^2^ values the easier to see it. This periodicity may appear or disappear by simple random fluctuations; so the sign of 0 is ±. AA pairs that did not presented a sign periodicity of deviations from randomness in Table 1 show a tendency to a ++ − cycle in FD and a definite ++ − periodicity with SD. AT pairs together with TA pairs showed the smallest χ^2^ values and did not presented a clear periodicity in Table 1, but they presented a visible periodicity + −− or ++ − in Table 3 that is interrupted by non periodical elements that, with a few exceptions, are very low values. The AG and AC pairs without periodicity in their sign of deviation from randomness in Table 3 showed in their χ^2^ FD and SD impressive ++ − periodicities. The same occurred for TT and TG pairs with ++ − or + −− periodicities. In Table 4 where G and C are the first nucleotide of the pair the rule of periodicities ++ − or + −− found in FD or SD is applied throughout the series with few exceptions (most of them in relation to low significant values). Thus our first aim is satisfied: the structure of the periodicity is due to the positive (attraction) or negative (repulsion) strength of association of both bases of the 16 dinucleotides that varies periodically according to the 3K period of site separations. Neither periodic sequence, nor latent periodicities were needed to elucidate this mtDNA periodicity. Since it is difficult to see the periodicities from Tables 3 and 4, the Additional file 1 is provided. We see that complete 3K periodicities of signs (+ − +) were seen in AC, TG, GT, GG, CA and CC pairs. These pairs show seven triplets in tandem. It is to be remarked the complementary (3′-5′ and 5′-3′) nature of AC-TG-GT-CA and GG-CC pairs (see analysis IV of complementary correlations below). Several other repeats are seen in the other pairs but they are incomplete in the whole series.

### Discarding contiguousness

A critical view to these periodicities may arise from the large significant association between contiguous bases: if a base in the site n_th_ associates to the base in site (n + 1)_th_, and the base in the site (n + K)_th_ associates to the base in the site (n + K + 1)_th_, then we should find an association when studying bases separated by K sites (we named this condition as contiguousness). This is true if and only if the association structure is isotropic along with the whole DNA that is, the influence of contiguousness is the same between sites n_th_-(n + 1)_th_ and (n + K)_th_-(n + K + 1)_th_ (the neighbor influence hypothesis); however, this condition is not necessarily true. We devised a test to rule out contiguousness by choosing the first base every I sites and the second one J sites downstream (we used I = J, but they may be different). With this method we destroy the possibility of contiguousness, the neighbor influence of bases [35–37] and any periodic sequence. Table 5 presents this analysis until I = J = 9 for A and T and until I = J = 15 for G and C. For A, the last clearly significant individual result was found at I = J = 6; if we take a pair A-A where the first A is taken at least every 6 sites and the second A happens 6 sites downstream, we found that there are more A-A (χ^2^_1_ = 4.5) and less A-G (χ^2^_1_ = 13.4) than expected; other significant values appeared at 52–52 (A-G: χ^2^_1_ = 4.6), 54–54 (A-G: χ^2^_1_ = 4.1) and 76–76 (A-G: χ^2^_1_ = 4.1). For T, as the first base, the last highly significant value is found at I = J = 3 where T-G pairs happened less frequently than randomly expected (χ^2^_1_ = 8.5); a less significant value happened at 76–76 (T-G: χ^2^_1_ = 4.8). It was remarkable that in these 4 last significances the second base was G. For G as the first base we found significant values until 66–66 (G-C: χ^2^_1_ = 9.5) and 72–72 (G-A, χ^2^_1_ = 4.3). For C a significant result was found at 60–60 (C-G: χ^2^_1_ = 8.6). As in Tables 1 and 2, G and C are involved in the strongest interactions and periodicities. It is very probable that periodicities and interactions happen between bases separated by larger Ks, but a larger genome is needed to show that [8, 9]. If we consider the largest significant Sep at 76–76 it involves only 19,517/76 = 257 pairs (near 16 expected pairs for each of the 16 pairs) and significance for the 16 different pairs is not an expected result with the χ^2^_1_ test. We found significant values over 100–100 Sep with complete bacterial genomes and significant selective interactions with human chromosome 21 [8, 9] whose significant interactions were found until K = 15,000,000.

**Table 5:** **Periodical selective deviation of dinucleotides measured by the χ** ^**2**^_**1**_ **test contribution when the first base is taken every I sites and the second J sites downstream**

1° B		Adenine				Thymine			
2° B		A	T	G	C	A	T	G	C
I-J	Tχ^2^_9_	Contribution of pairs (χ^2^_1_) to the total χ^2^_9_ value							
2-2	42.3	0.5+	0.1+	5.2-	0.0+	0.3-	1.2+	0.0+	1.5-
3-3	171.5	1.1+	1.7+	4.1-	8.2-	2.5+	0.4+	8.5-	3.6-
4-4	33.8	3.1+	5.4-	2.2+	0.1-	0.6-	3.5+	0.6-	1.7-
5-5	17.9	0.8-	1.7+	0.6-	0.0-	0.0-	0.0+	0.3-	0.4+
6-6	106.8	4.5+	0.3+	13.4-	4.2-	0.0-	0.2+	1.1-	0.1+
7-7	18.1	0.8-	0.7+	0.2+	0.0-	2.3+	1.3-	0.0+	0.8-
8-8	16.1	1.0+	0.0+	0.3-	3.3-	0.2-	0.2+	0.7-	0.5+
9-9	51.8	0.4+	1.0+	3.8-	2.0-	1.8+	0.3-	1.2-	0.5-
**1° B**		**Guanine**				**Cytosine**			
2-2	42.3	0.4+	0.1-	0.2+	0.9-	0.6-	6.2-	15.1+	10.0+
3-3	171.5	5.5-	5.0-	48.1+	8.8+	10.0-	3.6-	13.4+	47.1+
4-4	33.8	4.8-	0.8+	0.6+	4.1+	0.0-	0.1+	4.6-	1.9+
5-5	17.9	2.6+	2.9-	2.9+	1.9-	0.6+	1.9-	1.3+	0.0+
6-6	106.8	6.5-	2.4-	54.3+	2.2+	3.8-	0.2-	8.1+	5.6+
7-7	18.1	2.7-	0.2+	0.1-	8.2+	0.1+	0.1+	0.5-	0.3-
8-8	16.1	0.1-	0.2-	0.4+	1.0+	0.8-	0.6-	4.6+	2.2+
9-9	51.8	4.2-	0.2-	13.6+	3.5+	4.1-	0.3-	7.9+	7.1+
10-10	14.6	1.7-	0.1+	0.0+	3.8+	0.2-	1.6+	0.0-	2.9-
11-11	13.0	0.3+	0.1-	0.0+	0.4-	3.2-	0.2+	7.2+	0.3+
12-12	52.2	8.6-	0.0-	15.1+	5.8+	0.6-	1.5-	1.9+	7.0+
13-13	27.1	3.5-	0.0+	0.3-	15.2+	1.2+	2.0-	0.1-	0.6+
14-14	14.2	0.0+	1.1+	1.4-	1.3-	3.6-	0.5+	4.9+	0.4+
15-15	33.8	1.2-	0.6-	0.3+	9.6+	3.1-	0.2-	6.3+	4.3+

### Discarding sequence periodicities or latent periodicities

In Introduction we established conclusive differences between these stochastic periodicities of the distance to random distribution of dinucleotides with sequence periodicities studied by Fourier’s analyses, autocorrelations, latent periodicities (textual strings) or other methods. The present results add more conclusive proofs to this difference. 1) The structure of these stochastic periodicities reveals that it is due to the periodic variation of the strength of non-random association of both bases of dinucleotides separated by 1, 2, 3, …K nucleotide sites which is present anywhere in the whole mtDNA and it is not due to periodic repeats of DNA sequence. 2) Discarding contiguousness is equivalent to discard sequence periodicity; if the first base jumps every I number of sites and the second base is taken every J number of sites downstream any repeat of a sequence periodicity should be destroyed. We know that mtDNA has weak sequence periodicities in tDNA, rDNA, mDNA (for coding subunits of proteins with the same evolutionary origin); but it is difficult to ascertain them with current tests, because the small number of units sharing these periodicities; they are latent (not visible) periodicities and need sophisticated tests to be seen. So, it is expected that significance of these tests, applied to this mtDNA, should be moderate or none. The fact that we are dealing with a very different kind of periodicities is demonstrated by the huge values of significance that can be seen for any researcher with our simple tests. In fact they were discovered by under-graduated students in their training for using statistical tests in genome sequences. They are not latent, in the current English language; they are evident, apparent and directly ascertained. Several significant values occur with probabilities lower than 10^−20^ for the set of pairs or for individual pairs. Another, important difference is that they are present along with DNA segments or in whole genomes, regardless that the first base belong to one kind of DNA and the second to the same or another kind of DNA. Moreover, the significance of these stochastic periodicities decreases as K increases, the significance of sequence periodicities remain stable along with the genome.

### Other criticisms and the expected bases’ complementariness periodicity

Other critical views can arise from other biologic, mathematical and statistical restrictions (4 bases, assumption of randomness, neutrality of the genomes, and so on) but they are out of the scope of this article. We cannot completely rule out the possibility that periodicities may be produced by complex hidden methodological artifacts; this possibility is highly improbable with the last described tests and the expected resistance to base alterations. We have a powerful tool to test artifacts with the condition of equal association behavior of both bases in both senses and both strands due to complementariness. If our discovery that there is a tendency to a periodic strength of non-random association is true we can test it with DNA complementariness. As for example T-C in strand S1 and sense 5′-3′ is complementary with the pair G-A in strand S2 and the same sense 5′-3′, but it is complementary with the pair A-G in S2 and sense 3′-5′; thus we can study in S1 the correlation of interactions and periodicities of T-C with G-A and A-G. To describe all these possible correlations we studied the Pearson’s correlation coefficient for the 22 values of the χ^2^_1_ among the 16 pairs presented in Tables 1 and 2. Table 6 presents these values where Sep 1 and Sep 2 have been excluded because they are surely biased by contiguousness. Table 6 shows 120 correlation values, from which, at random, 6 should be significant at the 0.05 level, 1.2 at the 0.01 level and 0.12 (none) at the 0.001 level. With a two tailed t test and 20 degrees of freedom (22–2) we found 25 significant correlations at the 0.05 level, 14 at the 0.01 level, 23 at the 0.001 level and 58 non-significant correlations. This result is conclusive for the biotic meaning of these periodic deviations from randomness. The 16 pairs can be tested for the complementariness test from this panel of correlations; this analysis is presented in Additional file 2. Equal pairs are expected from complementariness (AT-AT, CG-CG, etc.), but most independent correlations were highly significant and positive, four of them occur with probabilities less than 10^−6^. Two negative independent correlations were non-significant and one of them include the C-G pair that have a known particular regulatory behavior when cytosine is methylated (the 3′-5′ sense is relevant); this does not occur only in contiguous CpG pairs (in this analysis are excluded) but, it seems it is a property of methylated cytosine [38–40].

**Table 6:** **Correlations coefficients of the χ** ^**2**^_**1**_ **contribution of the 16 pairs shown in Tables** 3 **and** 4 **for 22 values (excluded Sep 1 and 2)**

DN	AA	AT	AG	AC	TA	TT	TG	TC	GA	GT	GG	GC	CA	CT	CG
AT	*-.56*														
AG	**-.47**	-.28													
AC	**-.52**	-.07	*.65*												
TA	*-.57*	***.66***	.10	.01											
TT	***.93***	*-.63*	-.24	-.33	*-.55*										
TG	-.23	-.10	.12	*.60*	-.21	-.22									
TC	-.05	.03	-.25	.11	-.37	-.17	**.48**								
GA	**-.51**	.07	.32	*.63*	-.14	**-.46**	**.51**	.40							
GT	**-.50**	-.18	***.67***	***.87***	-.10	-.41	**.46**	.09	***.75***						
GG	**.51**	.16	***-.67***	***-.88***	.12	.39	*-.59*	-.07	***-.79***	***-.95***					
GC	.40	-.18	-.04	-.39	.20	**.43**	-.23	**-.53**	***-.84***	*-.57*	**.48**				
CA	*-.57*	-.11	*.65*	***.87***	-.10	-.42	***.74***	.22	***.76***	***.84***	***-.92***	**-.45**			
CT	-.20	-.33	.42	.40	.01	-.25	**.46**	-07	.13	**.45**	**-.47**	.16	**.47**		
CG	**.43**	.34	***-.80***	**-.49**	-.03	.25	**-.44**	.10	-.26	**-.49**	*.60*	-.12	***-.71***	*-.63*	
CC	**.45**	.08	**-.45**	***-.82***	.11	.42	***-.76***	-.35	***-.68***	***-.81***	***.85***	**.43**	***-.86***	*-.63*	**.50**

DN = dinucleotide. The correlations between equal pairs are not presented because they are always 1.00. Statistical significance at the 0.05 level is found with a correlation coefficient value = ± .424. Two tailed significance: **0.XX** = at the 0.05 level; *0.XX* = at the 0.01 level; ***0.XX*** = at the 0.001 level.

### Non random internucleotide interactions, periodicities and evolutionary theories

It is important to well understand the selective nature of periodicities and interactions. Table 7 shows the relative fitness and selection coefficient for the 16 pairs when Seps are 12–12 that is the first base was chosen every 12 sites and the second 12 sites downstream. For example, the first pair is 1st – 13th, then 12th -24th, 24th -36th and so on; if the first nucleotide is the 2nd one of the mtDNA, values change a little but the 3-sites periodicity is conserved; this sampling yields 1,626 pairs. The mutation rate of mtDNA is near 10^−6^ mutant-base/(cycle of replication) that is, it is expected a change of base at any site, as an average, every million mitochondrion cycles (homologizing rates to those of prokaryotes and viruses [41–43]). There may be two, three or more mtDNA cycles a day, thus, 1,000 cycles a year is a reasonable figure. In 1,000 years there are one million cycles, the time for one change of base in a site. However, this mtDNA of *Drosophila melanogaster* has remained like the present DNA for hundred thousand years or more. Let us examine the situation in 100,000 years, where 100 changes are expected at every site. The expected random distribution of these 100 mutants is given by the frequency of bases we found in the whole DNA. Let us examine in Table 5 the case of G as the first nucleotide (I = J = 12, it is mentioned in Methods). The expected frequencies for the second base are: fA = 0.4177; fT = 0.4039; fG = 0.0758; fC = 0.1026 as we calculated previously. These frequencies give the random expected number of pairs (of Table 7) whose first base is G : 58.5G-A; 53.0G-T; 11.7G-G; 14.8G-C; however, we found 36, 53, 25 and 24 pairs, respectively. Among 100 expected mutant pairs produced in these 100,000 years there are 41.7G-A, 40.4G-T, 7.6G-G and 10.3G-C; but the observed pairs should have been 26, 39, 18 and 17, respectively. During this time 15.7G-A and 1.4G-T pairs were negatively selected, and 10.4G-G and 6.7G-C pairs were positively selected; these selective conditions have been maintained during these 100,000 years. Thus the relative, to non-selected pairs (random produced or expected pairs), fitness is obtained by the quotient between the observed and the expected number of pairs, and the relative selection coefficient (RSC) is obtained by subtracting this relative fitness to 1. We observe, in Table 7, that RSCs move between −0.38 and +1.14 and only one is near 0 (G-T: Expected 53.04428, Observed 53, χ^2^_1_ Con = 0.00003687, R fitness = 0.99917, RSC = −0.000835). It is to be remarked that all these calculations are performed by assuming this mtDNA (with its base frequencies) has fitness 1, but this is highly probable false, because for a population of flies to subsist, among a great deal of negative contingencies that kill a lot of flies regardless their genomes, it is needed that individual genomes have an overall fitness far over 1. These fitness and selection coefficients are only compatible with the Synthetic Theory of Evolution [1, 4–8].

**Table 7:** **Relative fitness and selection coefficient of the 16 dinucleotides when their first base was chosen every 12 sites and their second one was chosen 12 sites downstream**

Pair	Expected	Observed	Con χ^2^_9_	R Fitness	RSC
A-A	292.4	305	0.5447	1.04	+ 0.04
A-T	265.2	285	1.4750	1.07	+ 0.07
A-G	58.6	44	3.6205	0.75	- 0.25
A-C	73.8	56	4.3092	0.76	- 0.24
T-A	264.4	281	1.0405	1.06	+ 0.06
T-T	239.9	230	0.4047	0.96	- 0.04
T-G	53.0	49	0.2960	0.92	- 0.08
T-C	66.8	64	0.1153	0.96	- 0.04
G-A	58.5	36	8.6389	0.62	- 0.38
G-T	53.0	53	0.0000	1.00	- 0.00
G-G	11.7	25	15.0754	2.14	+ 1.14
G-C	14.8	24	5.7720	1.62	+ 0.62
C-A	73.7	67	0.6144	0.91	- 0.09
C-T	66.9	57	1.4601	0.85	- 0.15
C-G	14.8	20	1.8540	1.35	+ 0.35
C-C	18.6	30	6.9552	1.61	+ 0.61
Total	1626.1	1626	52.1761		

Con = χ^2^_1_ contribution to; R fitness = relative fitness obtained by Observed/Expected (where expected fitness = 1); RSC = relative selection coefficient (R Fitness – 1).

We have discovered a periodicity of the χ^2^ value that measures the non-random association of the two bases of dinucleotides whose nucleotides are separated by 0, 1, 2…K nucleotide sites. This stochastic periodicity (the variable that is periodic is the χ^2^ value that is a random variable) is different to previous periodicities based on DNA sequences (the variable that is periodic is a nucleotide sequence) where the χ^2^ or other tests are used to estimate the significance of the existence of a sequence periodicity. In our periodicity the χ^2^ value of non-randomness constitutes by itself or describes directly the periodicity. As the χ^2^ value measures non-random association of both bases of dinucleotides, it measures the strength of positive (more than randomly expected) and negative (less than randomly expected) tendency for their association according to the number of sites between them. As we have assumed that the neutral (random) expected frequencies of bases fA, fT, fG, fC are those find in the chromosome or DNA segment, and with these frequencies we estimated the random (neutral) expectancy of each of the 16 possible dinucleotides, the deviations from this expectancy measured by the χ^2^ value is also the measure of the natural selection that operated in this chromosome during the time it has remained so constituted. This mtDNA has remained like that for more than one million of cell generations during which this enormous selective pressure has operated, a process that can be only understood within the STE.

We found that the stochastic periodicity is present in the 16 pairs regardless their distribution or allocation, thus any of them presents periodicity of the strength of their bases’ association. As for example, C in the CG pairs associates with G periodically where the strength of association, in relation to the expected random association, varies with the number of sites between both when these numbers are (3K-1), (3K) and (3K + 1). This occurs with the 16 pairs with different significance; it is more significant when C and G are involved; the smallest significance is found in AT and TA pairs. Our aim to find the tip of the thread has been accomplished. Any base interacts non-randomly with the remaining bases of the chromosome; these interactions include a 3K periodic component as far as some prokaryote genomes and this mtDNA are concerned.

Criticisms due to the big non-random association of contiguous bases and neighbor influences of bases have been discarded by taking the first base every I sites and the second base J sites upstream. After this procedure significant periodicities were apparent until I = J = 76 when 257 pairs yielded a significant deviation from randomness. This procedure additionally discards the possibility of sequence periodicities as the main factor of these periodicities and refutes the hypothesis of neighbor influences of bases. These analyses show a sharp difference with those performed to evidence cryptic, hidden, invisible or latent periodicities; stochastic periodicities are apparent at the first sight with significance probability often lower than 10^−20^, even though this is a small DNA with less than 20,000 bp.

The most relevant result is the interaction of any base of the genome with the set of the other bases (the residual genome). Our vision of selection must shift from considering the environment as the most important element of selection to the residual genome as an internal main factor for co-adaptive selection and evolution (an inner residual genome environment? Self-genome selective processes? If so, conservation is much more important than variation in evolution).

## Conclusions

We add strong evidence on the non-random interaction between both bases of dinucleotides (pairs) separated by 0, 1, 2 … K nucleotide sites, and for the selective 3K periodicity of the χ^2^ to measure the distance to randomness of dinucleotide distribution in the *D melanogaster* mtDNA. These interactions and periodicities indicate that any base in this genome is co-adapted with every base of the residual genome, a condition only compatible with the Synthetic Theory of Evolution and agrees with the Wright’s adaptive landscape where evolution implies shifting of resilient adaptive peaks. We describe the structure of the 3K periodicities for the 16 dinucleotides; pairs where G and C are involved are more distant from randomness than pairs where A and T are involved. This structure also shows that sequence periodicities are not the cause of these selective periodicities and several other evidences of the difference between both types of periodicities are given. Sampling pairs when the first base is chosen every I sites and the second is taken J sites downstream ruled out the contiguousness condition that may explain these interactions and periodicities, and also sequence periodicities. Correlations between the χ^2^ values among the 16 pairs considering the complementary condition of both DNA strands discarded most non-biotic origins of these interactions.

## Methods

In this article the complete mtDNA of *Drosophila melanogaster* obtained from GenBank [ACN = NC 001709, with 19,517 nucleotide sites; 8,152A (41.77 %); 7,883 T (40.39 %); 1,479G (7.58 %); 2,003C (10.26 %)] was analyzed. N will be the total number of sites. With four bases, A, T, G and C 16 dinucleotides or pairs are possible. We could not find analyses of sequence periodicities for mtDNAs, in the literature, even though they do exist because mtDNAs have coding regions for tRNAs, rRNAs and other for subunits of proteins with a common evolutionary origin even with bacteria (endosymbiotic origin of mitochondria). However we published the total base colored map of this mtDNA [6] and no evident periodicity was observed excepting a small segment of TATATA… tandem. We assume that the random expected proportion of bases is that given by their present frequency in this mtDNA: fA = 0.4177; fT = 0.4039; fG = 0.0758; fC = 0.1026. The product of two base frequencies gives the random expected pair frequency. For example f(A-G) = fAxfG = 0.4177 × 0.0758 = 0.03166. The expected number of each pair is obtained by multiplying its frequency by the number of pairs. We studied dinucleotides whose bases were separated by 0 (contiguous bases), 1, 2, 3, … K sites. Any K series can be thought as a window of (K-1) sites flanked by the first nucleotide (base) at the left end and by the second nucleotide at the right end that moves scanning the whole genome collecting dinucleotides so constructed. With 19,517 bases we can obtain 19,516 pairs with contiguous bases (0 site separation), 19,515 pairs with bases separated by 1 site, 19,514 pairs with bases separated by 2 sites and (N - K - 1) pairs with bases separated by K sites. The restriction in the number of bases is due to our work with linear mtDNA; the circular mtDNA does not have this restriction, but it is not the present aim of this study. To interpret these values we need to know that the significant critical values of the χ^2^_9_ test [the χ^2^ with 9 degrees of freedom, given by four (less one) times four (less one) bases to yield the table of 16 pairs] for probabilities 0.05, 0.025, 0.01, 0.005 and 0.001; they are 16.9, 19.0, 21.7, 23.6 and 27.9, respectively; thus, 17 or more is considered a significant result (at least at the 0.05 level). To give significant values to the deviation of each pair we used the χ^2^_1_ contribution to the total χ^2^_9_ value; their critical value is 3.84, 5.02, 6.64, 7.88 and 10.83 for the probabilities 0.05, 0.025, 0.01, 0.005 and 0.001 respectively; thus over 3.84 we can consider a significant pair deviation from randomness. It is important to remark that the χ^2^ contribution to the χ^2^_9_ is not the total value of the χ^2^_1_ test, because it misses the complement of this pair contribution to the total. However, this remaining contribution to the χ^2^_1_ test is much smaller than the contribution of the pair, and it will be omitted. As for example, one expected value of G-G is 11.7122 (when the first base was chosen every 12 sites and the second one 12 sites downstream, see results) and the corresponding observed value is 25 in 1,626 pairs; the contribution of the pair is (25–11.7122)^2^/11.7122 = 15.075; for the total χ^2^_1_ it needs the addition of the contribution to the complement to the total (expected value is 1,626 – 11.7122 = 1,614.2878 and the observed one is 1,626 – 25 = 1,601) that is (1,601 – 1,614.2878)^2^/1,614.2878 = 0.1094, which is much smaller than 15.075 and may be disregarded without a substantial error.

A very important methodological feature should be remarked on both types of periodicities. Previously described sequence periodicities are greatly depending on deletions or insertions of bases, inversions, missing base information, miss-assignment of bases or other informational errors. These alterations of the sequence period destroy the period or the concordance with the textual string used to detect them. Our stochastic periodicities are almost insensible to them. For our dinucleotide series (remember the analogy of the window scanning the genome) all the dinucleotides whose both bases are located upstream or downstream the alteration will result unchanged. Only the dinucleotides whose first base precedes the alteration and whose second base follows the alteration will change; this change will be moderate or small due to contiguousness (contiguous bases tend to be the same, AA, TT, GG and CC are the most frequent pairs [4–6] and because this may or may not change the stochastic χ^2^ period according to the base composition included in the segment of K sites, and because there is a great correlation of dinucleotides reading them up or downstream (see Results).

Another very important difference is that fixed sequence (previous) periodicities with period different from 3K (the period found in stochastic periodicities) shall introduce a big noise in the stochastic periodicity with period = 3. Sequence periodicities, with periods different from 3K, contribute enormously to non-random, interactions that can blur this 3K periodicity. Besides that the coding structure for non periodic RNA or proteins is another enemy of stochastic periodicities. This is, perhaps, the cause of our present failure to find these periodicities in segments of eukaryote DNA (it is expected an average of periods of STRs, VNTRs, LINES, SINES, oncogenes, transposons and so on), but it is expected that a long DNA segment (a chromosome) shows some periodicity as and average as we have recently found [8, 9]. We must remember that our periodicity is one of the two stochastic components of the value to measure internucleotide interactions with a periodical and a non(a)-periodical component. Since both components vary stochastically the a-periodical component may (and very often does) blur or hide the periodical one.

## Electronic supplementary material

Additional file 1: **Regular recurrent pattern of 3 sites of sign repeats that appear when the first or second derivative of the χ** ^**2**^_**1**_ **value is obtained from Tables** 3 **and** 4 . (DOC 34 KB)

Additional file 2: **Observed correlations of dinucleotide periodicities of pairs in the cis-strand deduced from the complementary pairs in the trans-strand in both 5′-3′ and 3′-5′ sense.** (DOC 39 KB)
