# Supplementary material for: The structure of selective dinucleotide interactions and periodicities in D melanogaster mtDNA
Source: Biol Res. 2014 May 23;47(1):18. doi: 10.1186/0717-6287-47-18 (PMC4101722; doi:10.1186/0717-6287-47-18)
Supplement: Supplementary file 1 — Additional file 1: Regular recurrent pattern of 3 sites of sign repeats that appear when the first or second derivative of the χ 2 1 value is obtained from Tables 3 and 4 . (DOC 34 KB) [file 40659_2014_9_MOESM1_ESM.doc]

**Additional file 1**

**Regular recurrent pattern of 3 sites of sign repeats that appear when the first or second derivative of the χ^2^_1_ value is obtained from Tables 3 and 4.**

**K**

**DN De 2 3 4 5 6 7 8 9 10 11 12 13 14 15 16 17 18 19 20 21 22 23 24**

**AA 2° + + - + - + + - + + - + + - + + - + + - + +**

**AT 2° + + - + + - + - + - + + - + - + - - + - - +**

**AG 1° + - - + + - + + - + + - + + - + + - + + - + +**

**AC 2° + - + + - + + - + + - + + - + + - + + - + +**

**TA 2° + + - + + - + - + - + + - + + + - + + - + -**

**TT 2° + + - + + - + + + + - + + - + + - + + - + +**

**TG 2° + - + + - + + - + + - + + - + + - + + - + +**

**TC 2° + - + + + - + + - + - - + - - + - - + - + +**

**GA 2° + - + + - + + - - + - - + - + + - - + - + +**

**GT 2° + - + + - + + - + + - + + - + + - + + - + +**

**GG 2° + - + + - + + - + + - + + - + + - + + - + +**

**GC 2° + - - + - - + - - + - - + - - + - - + - + +**

**CA 2° + - + + - + + - + + - + + - + + - + + - + +**

**CT 2° - - + - - + - + + - - + + - + + - + + - + -**

**CG 2° - - + - - + + - + - - + - - + - - + + - + +**

**CC 2° + - + + - + + - + + - + + - + + - + + - + +**

**DN = dinucleotide; De = discrete derivative; 3-sites periods underlined.**
