# Supplementary material for: The structure of selective dinucleotide interactions and periodicities in D melanogaster mtDNA
Source: Biol Res. 2014 May 23;47(1):18. doi: 10.1186/0717-6287-47-18 (PMC4101722; doi:10.1186/0717-6287-47-18)
Supplement: Supplementary file 2 — Additional file 2: Observed correlations of dinucleotide periodicities of pairs in the cis-strand deduced from the complementary pairs in the trans-strand in both 5′-3′ and 3′-5′ sense. (DOC 39 KB) [file 40659_2014_9_MOESM2_ESM.doc]

Additional file 2

Observed correlations of dinucleotide periodicities of pairs in the cis-strand deduced from the complementary pairs in the trans-strand in both 5’-3’ and 3’-5’ sense.

OPCS SS-CP r Prob DS-CP r Prob

AA TT(+) 0.9307 <10^-6^ TT

AT AT TA 0.6596 0.0008

AG CT(+) 0.4196 0.0516 TC(-) -0.2483 0.2653

AC GT(+) 0.8679 <10^-6^ TG(+) 0.5968 0.0034

TA TA AT(+) 0.6596 0.0008

TT AA(+) 0.9307 <10^-6^ AA

TG CA(+) 0.7386 0.0001 AC(+) 0.8691 <10^-6^

TC GA(+) 0.4010 0.0641 AG(-) -0.2483 0.2653

GA TC(+) 0.4010 0.0641 CT(+) 0.1281 0.5700

GT AC(+) 0.8679 <10^-6^ CA(+) 0.8400 <10^-6^

GG CC(+) 0.8500 <10^-6^ GG

GC GC CG(-) -0.1170 0.6041

CA TG(+) 0.7386 <10^-6^ GT(+) 0.8400 <10^-6^

CT AG(+) 0.4196 0.0516 GA(+) 0.1281 0.5700

CG CG GC(-) -0.1170 0.6041

CC GG(+) 0.8500 <10^-6^ CC

OPCS = original pair in the cis-strand. SS-CP = same sense pair in the trans-strand correlated in the cis-strand. DS-CS = different sense pair in the trans-strand correlated in the cis strand. r = Correlation coefficient. Prob = Probability with the t test and 20 degrees of freedom.
